# The Prevalence of Metabolic Syndrome and Its Components among People with Type 2 Diabetes in the Ho Municipality, Ghana: A Cross-Sectional Study

**DOI:** 10.1155/2017/8765804

**Published:** 2017-02-12

**Authors:** James Osei-Yeboah, William K. B. A. Owiredu, Gameli Kwame Norgbe, Sylvester Yao Lokpo, Jones Gyamfi, Emmanuel Alote Allotey, Romeo Asumbasiya Aduko, Mark Noagbe, Florence A. Attah

**Affiliations:** ^1^Department of Medical Laboratory Sciences, School of Allied Health Sciences, University of Health and Allied Sciences, Ho, Ghana; ^2^Department of Molecular Medicine, School of Medical Sciences, Kwame Nkrumah University of Science and Technology, Kumasi, Ghana; ^3^Department of Clinical Biochemistry, Diagnostic Directorate, Komfo Anokye Teaching Hospital, Kumasi, Ghana; ^4^School of Allied Health Sciences, University of Health and Allied Sciences, Ho, Ghana; ^5^Laboratory Department, Ho Municipal Hospital, Ghana Health Service, Ho, Volta Region, Ghana; ^6^Diabetic Clinic, Ho Municipal Hospital, Ghana Health Service, Ho, Volta Region, Ghana

## Abstract

The cooccurrence of diabetes mellitus and metabolic syndrome potentiates the cardiovascular risk associated with each of the conditions; therefore characterizing metabolic syndrome among people with type 2 diabetes is beneficial for the purpose of cardiovascular disease prevention. This study aims at evaluating the prevalence of metabolic syndrome and its components among 162 patients with type 2 diabetes attending the diabetic clinic of the Ho Municipal Hospital, Ghana. Data obtained included anthropometric indices, blood pressure, serum lipids, glucose, and sociodemographics and clinical information. The overall prevalence of metabolic syndrome among the study population was 43.83%, 63.58%, and 69.14% using the NCEP-ATP III, the WHO, and the IDF criteria, respectively. The most predominant component among the study population was high blood pressure using the NCEP-ATP III (108 (66.67%)) and WHO (102 (62.96)) criteria and abdominal obesity (112 (69.14%)) for IDF criteria. High blood pressure was the most prevalent component among the males while abdominal obesity was the principal component among the females. In this population with type 2 diabetes, high prevalence of metabolic syndrome exists. Gender vulnerability to metabolic syndrome and multiple cluster components were skewed towards the female subpopulation with type 2 diabetes.

## 1. Introduction

There is a growing interest in a cluster of synergistically interacting cardiovascular risk factors called metabolic syndrome (MetS) [1]. The syndrome has also been described as Insulin Resistance Syndrome, Deadly Quartet, and Syndrome X [2, 3]. The main components associated with the metabolic syndrome include raised blood pressure, dyslipidaemia (raised triglycerides and lowered high density lipoprotein cholesterol), raised fasting glucose, and central/abdominal obesity [1, 4].

Metabolic syndrome is an extremely important parallel risk contributor to heart disease [5]. Isomaa et al. [6] reported a threefold increased risk of coronary heart disease and stroke, as well as markedly increased cardiovascular mortality among subjects with metabolic syndrome. There is a surge in prevalence of many of the attributable components of MetS, causing a worldwide pandemic with implication for both clinical and public health [7]. The available evidence indicates that in most countries, 20% to 30% of the adult population can be characterized as having MetS [7–9].

In Ghana, various studies among various subpopulations have reported different prevalence rates based on different definitive criteria. Among them, Gyakobo et al. [3] reported a prevalence of 35.9% using the International Diabetes Federation (IDF) criteria and 15.0% using the National Cholesterol Education Program Adult Treatment Panel III (NCEP-ATP III) in a rural community. Kow Nanse Arthur et al. [10] reported a prevalence of 14.4%, 25.6%, 29.2%, and 30.4% using the World Health Organization (WHO) criteria, the NCEP-ATP III criteria, the IDF criteria, and the Harmonised Metabolic Syndrome (H_MS) criteria, respectively, among adult females.

Diabetes mellitus is a group of metabolic diseases that is associated with disturbance in the body's metabolism and energy utilization from carbohydrates, lipids, and proteins [11, 12]. Patients with diabetes are prone to complications driven by both modifiable and nonmodifiable risk factors [13]. A strong association between MetS and incident diabetes exists [14]. The cooccurrence of diabetes mellitus and MetS potentiates the cardiovascular risk associated with each condition [15]. Some investigators favour keeping the risk factors separated for purposes of clinical management, whereas others believe that identifying individuals with an aggregation of risk factors provides additional useful information to guide clinical management [4, 16]. Thus characterizing MetS among patients with diabetes is beneficial for multiple risk reduction including lifestyle factors with the ultimate purpose of preventing cardiovascular disease [4, 16].

Among the Ghanaian population with diabetes, predominantly high prevalence of MetS (24–78.8%) has been reported at different locations, with different definitive criteria [1, 13, 17–19]. Due to the influence of genetic and lifestyle factors in the development of MetS, the prevalence and principal components of the syndrome vary among populations [20]. There is a dearth of information in the Ghanaian literature on metabolic syndrome among people with type 2 diabetes within the regional jurisdiction of the current work (Volta region). Using three definitive criteria (NCEP-API III, WHO, and IDF), this study is aimed at evaluating the prevalence of metabolic syndrome (MetS) and its component among people living with type 2 diabetes in the Ho Municipality.

## 2. Materials and Methods

### 2.1. Sampling

A hospital-based cross-sectional study was conducted between February 2016 and April 2016. One hundred and sixty-two (162) previously diagnosed patients with type 2 diabetes attending diabetic management clinic at the Ho Municipal Hospital in the Volta Region, Ghana, were conveniently and purposively recruited for this study. The study populations were of consent age (25–86 years) and were all living in the Ho Municipality.

### 2.2. Sociodemographic Data Capture (Questionnaire)

Self-reported semistructured questionnaire was administered to determine duration of diabetes and treatment status, educational level, occupation, smoking status, alcohol intake, sugar intake, fat intake, and physical activity.

### 2.3. Haemodynamic Parameter

#### 2.3.1. Blood Pressure (BP) Measurement

After participants had sat quietly for at least ten minutes, their blood pressure (BP) measurements were done using the OMRON digital fully automatic blood pressure monitor (OMRON Healthcare, IntelliSense BP785, HEM-7222). Three measurements were taken using an appropriate sized cuff at one-minute interval on the right arm in a seated position, with the arm supported at heart level and feet flat on the floor [21].

#### 2.3.2. Anthropometric Measurements

Using a dual-purpose weight and height scale, body weight of participants was measured to the nearest 0.1 kg and height to the nearest 0.1 cm, with participants standing erect, back straight, heels together, feet slightly spread without shoes, and in light clothing (Health O meter® Professional Scales, 9500 West 55th St. McCook, IL 60525-7110, USA). Waist circumference (to the nearest centimeter) was measured with a Gulick II spring-loaded measuring tape (Gay Mills, WI) midway between the inferior angle of the ribs and the suprailiac crest. The hip circumference was measured as the maximal circumference over the hip circumference (HC) at the level of the widest diameter around the gluteal protuberance in centimeters. Body mass index (BMI) was calculated by dividing weight (kg) by the square of the height (m^2^). The waist-to-hip ratio (WHR) was calculated by dividing the waist circumference (cm) by the hip circumference (cm). Waist-to-height ratio was calculated by dividing the waist circumference (cm) by the height (cm). Other calculated adiposity indices were as follows:

*(1) Conicity Index (CI) [22]:*

(1) $$\begin{array}{c} CI=\frac{Waist\;\;Circumference\left(m\right)}{\left[0.109\times \sqrt{Weight\left(Kg\right)/Height\left(m\right)}\right]}. \end{array}$$

*(2) Abdominal Volume Index (AVI) [23]:*

(2) AVI=2Waist  Ccm2+0.7Waist  Ccm−Hip  Ccm21000.

*(3) Body Adiposity Index (BAI) [24]:*

(3) $$\begin{array}{c} BAI=\frac{Hip\;\;Circumference\left(cm\right)}{{\left[Height\left(m\right)\right]}^{1.5}}-18. \end{array}$$

*(4) Visceral Adiposity Index (VAI) [23]:*

(4) Males:  VAI=Waist  Circumferencem36.58+1.89×BMI×TG1.03×1.31HDL-CFemales:  VAI=Waist  Circumferencem36.58+1.89×BMI×TG0.81×1.52HDL-C.

### 2.4. Metabolic Syndrome Diagnosis Criteria

#### 2.4.1. National Cholesterol Education Program, Adult Treatment Panel III (NCEP-ATP III)

According to the National Cholesterol Education Programme, Adult Treatment Panel III (NCEP-ATP III), the criteria for the diagnosis of MetS include individuals with any three or more of the following five components: (1) abdominal obesity (waist circumference > 102 cm for men, or > 88 cm for women), (2) high triglyceride (TG) ≥ 1.7 mmol/L, (3) lowered high density lipoprotein cholesterol (HDL-C): men < 0.9 mmol/L or women < 1.0 mmol/L; (4) high blood pressure (BP) (systolic BP ≥ 130 mmHg or diastolic BP ≥ 85 mmHg or treatment of hypertension); and (5) high fasting glucose ≥ 6.1 mmol/L. However all participants were previously diagnosed patients with type 2 diabetes under management and, therefore, were classified as hyperglycaemic irrespective of the level of fasting blood glucose at the time of sampling [25].

#### 2.4.2. International Diabetes Federation (IDF) Criteria

According to the International Diabetes Federation (IDF), MetS can be diagnosed if central obesity (waist measurement > 90 cm for men or >80 cm for women) is accompanied by any two (2) of the following four (4) factors: (1) TG levels ≥ 1.7 mmol/L, (2) HDL-C < 1.03 mmol/L for men or <1.29 mmol/L for women, (3) high blood pressure (BP) of 130/85 mmHg or greater or treatment of previously diagnosed hypertension, and (4) a fasting blood glucose (FBG) of 5.6 mmol/L or greater or previously diagnosed type 2 diabetes [26].

#### 2.4.3. World Health Organization (WHO) Criteria

World Health Organization criteria also require the presence of diabetes mellitus, impaired glucose tolerance or insulin resistance, and any two of the following: (1) body mass index (BMI) ≥ 30 kg/m^2^ and/or waist-to-hip ratio >0.90 (male), >0.85 (female); (2) blood pressure ≥140/≥90 mmHg or on hypertension medication; and (3) triglyceride ≥ 1.7 mmol/L and/or HDL-C < 0.91 mmol/L (male), <1.01 mmol/L (female) [27].

### 2.5. Laboratory Assays

All serum biochemistry assays were carried out at the Ho Municipal Hospital Laboratory using the Random Access Fully Automated Vitalab Selectra Junior Chemistry autoanalyzer (Vital Scientific, BV Kanaalweg 24, Netherlands). After an overnight fast between 7 am and 10 am, about 5 mL venous blood samples was drawn from the antecubital vein of which four (4) milliliters was dispensed into a Vacutainer® serum separator tube using the closed vacutainer system and one (1) mL into fluoride oxalate tubes. The samples were centrifuged at 2500 rpm for 5 minutes to obtain the serum and plasma used for the assays. Using predetermined methods by the reagent manufacturer (ELITech Clinical Systems, SAS, Zone Indusrrielle-61500 SEES, France), the separated plasma and serum were used for the following assays: fasting blood glucose (FBG), total cholesterol (TC), triglyceride, and high density lipoprotein cholesterol. Very low density lipoprotein cholesterol (VLDL-C) and low density lipoprotein cholesterol (LDL-C) were calculated using the Fredrickson-Friedewald formula

(5) $$\begin{array}{c} \text{LDL-C}=TC-HDL-\frac{TG}{2.2}, \end{array}$$

where VLDL-C = TG/2.2.

### 2.6. Statistical Analysis

Normality of all continuous variables was tested. Continuous parametric variables were expressed as their mean ± standard deviation; continuous nonparametric variables were expressed as median (minimum and maximum), whereas categorical variables were expressed as frequency and proportion. Gender comparisons of parameters were performed using unpaired *t*-tests, Mann–Whitney *U* test, Chi square (*χ*^2^) test, or Fisher exact test where appropriate. A *p* < 0.05 was considered as statistically significant for all analyses. IBM Statistical Package for the Social Sciences version 22.00 was used for data analysis (SPSS Inc., Chicago, USA; http://www.spss.com).

## 3. Results

As shown in Table 1, one hundred and sixty-two (162) participants with a mean age of 56.42 ± 10.64 were recruited into this study. Sixty-one (37.65%) were males and 101 (62.35%) were females with matched average ages of 55.93 ± 11.25 and 56.71 ± 10.29, respectively. The majority of the study participants were married 119 (73.46%), with a higher proportion of the male participants reported married (56 (91.80%)) compared to their female counterparts (63 (62.38%)). A greater proportion (74.69%) of the study participants had attained at least secondary education at the time of this study. The majority (43.40%) of the study participants worked in the informal sector. Intake of dietary salt, sugar, and fat was generally low among the study population (see Table 1).

In general, the female participants presented with significantly higher anthropometric indices than their male counterparts. The exception, however, was seen with height where the males were averagely taller. Weight and waist-to-hip ratio were found to be comparable among the male and the female participants (see Table 2).

There was no significant gender difference in the levels of haemodynamic parameters measured for this study (SP and DP). Among the biochemical parameters assayed, the atherogenic lipid parameters such as total cholesterol, LDL-cholesterol, as well as triglyceride levels were observed to be significantly higher in the female population compared to the male population. Fasting blood glucose levels were found to be similar between both sexes (see Table 3).

The overall prevalence of metabolic syndrome among the study population was 63.58%, 43.83%, and 69.14% using the World Health Organization (WHO), the National Cholesterol Education Program, Adult Treatment Panel III (NCEP-ATP III), and the International Diabetes Federation (IDF) criteria, respectively. The prevalence was significantly higher among women using the WHO and the IDF criteria. However no statistical difference was observed between genders when the NCEP-ATP III criteria were used (Table 4).

Among the study population, there was no individual without a metabolic score, irrespective of the definitive criteria used. Also, there was no individual who had three or more metabolic syndrome scores but did not present with metabolic syndrome (see Table 4).

The prevalence of abdominal obesity was 48.15% using the NCEP-ATP III criteria and 69.14% using the IDF criteria. Using the WHO criteria, 30.86% of the study participants were classified as having central obesity. Abdominal/central obesity irrespective of the criteria used was found to be predominant among the female participants (Table 5).

Comorbidity of diabetes with hypertension ranged from 62.96% to 66.67% using the WHO and the IDF/NCEP-ATP III, respectively. No gender variation of diabetes with hypertension comorbidity was observed. Atherogenic levels of triglyceride were significantly tilted towards the female (see Table 5).

## 4. Discussion

The prevalence of metabolic syndrome (MetS) is increasing worldwide in both developing and developed countries [28, 29]. In the current study, the overall prevalence of metabolic syndrome observed among the study population was 43.83%, 63.58%, and 69.14% using NCEP-ATP III, WHO, and IDF criteria, respectively. Irrespective of the criteria used, there was no individual who possessed three or more metabolic scores and yet did not have metabolic syndrome. This is in agreement with earlier reports indicating that metabolic syndrome is a common phenomenon among the Ghanaian type 2 diabetes mellitus population. The prevalence of metabolic syndrome among people with type 2 diabetes in the Ghanaian population ranged within 43.3% NCEP-ATP III [19], 43.4% NCEP-ATP III, and 51.8% IDF [18] and 55.9% NCEP-ATP III [1], 58% NCEP-ATP III [13], and 78.8% WHO [18]. Similar reports in other jurisdictions of high prevalence rates of metabolic syndrome (63.6%, 51%, and 56%) have been recorded among people with type 2 diabetes, respectively, in North-Central Nigeria and Saudi Arabia [30–32]. The prevalence of metabolic syndrome was significantly higher in women than in men using the WHO and IDF criteria. The observation compares favourably with that of Titty et al. [1] and Nsiah et al. [13] where femininity accounted for higher metabolic syndrome scores. Similarly, a study in South-Western Nigeria reported a female preponderance to metabolic syndrome in a ratio of 3 : 2 to males among the people living with type 2 diabetes using the WHO criteria [32]. In contrast, observations in Jos, North-Central Nigeria, and the United States of America using the IDF definitive criteria revealed significantly higher occurrence of metabolic syndrome among the male subpopulation with diabetes compared to their female counterparts [30, 33]. The use of different diagnostic criteria for the definition of metabolic syndrome has been shown to account for the inconsistency in gender ratios observed in various populations [30].

The most predominant component of MetS abnormality among the study population was high blood pressure using the NCEP-ATP III (66.67%) and the WHO (62.96) criteria and abdominal obesity (69.14%) using the IDF criteria. Consistent with the findings of this study, Puepet et al. [30], Unadike et al. [34], and Nsiah et al. [13] have reported similar findings. Hypertension is identified as an independent cardiovascular risk factor and, in combination with insulin resistance, will have a synergistic effect which results in a grave risk for severe vascular events [34]. Abdominal/central obesity is associated with high amount of visceral fat, which is metabolically active, producing free fatty acids and inflammatory cytokines that drain directly into the liver via the portal circulation [20, 35]. This could elicit various complex injurious mechanisms leading to atherosclerosis and cardiovascular disease [35, 36].

Compared to previous studies in Ghanaian populations with diabetes, one of the strengths of the current study is the expanded scope of anthropometric parameters to include less commonly used candidate adiposity indices in this setting (Conicity Index, Abdominal Volume Index, Body Adiposity Index, and Visceral Adiposity Index). In general, the female subpopulation presented with significantly higher average anthropometric indices than their male counterparts (Table 2). The leading component of the MetS among the females with diabetes was abdominal/central obesity (Table 5). A previous study has recorded significantly higher measurements of obesity and adiposity among the females with type 2 diabetes compared to their male counterparts [16]. Though the reasons for this occurrence could not be directly inferred from the current study, gender disparities in obesity are influenced by acculturation through complex sociocultural pathways that are known to facilitate female weight gain in developing countries like those found in sub-Saharan Africa [37]. According to Nsiah et al. [13] women in Ghana are less likely to participate in physical exercises and live sedentary lifestyles than men and this may account for the odds of higher obesity and overweight.

Dyslipidaemia has been commonly demonstrated in subjects with metabolic syndrome [38]. The altered lipid levels associated with atherogenic dyslipidaemia are individual and collective risk factors for cardiovascular diseases [39]. Among the biochemical parameters assayed in this study, the atherogenic lipid parameters such as total cholesterol (TC), low density lipoprotein cholesterol (LDL-C), and triglyceride (TG) levels were found to be significantly higher among the female participants compared to their male counterparts (Table 3). This, however, contradicts the report by Gilani et al. [40]. In explaining the gender difference in lipid-lowering treatment outcome among the Chinese population, Zhang et al. [41] reported the biological baseline gender disparities in lipid levels as an attributed factor.

Though the mechanism of dyslipidaemia in persons with glycaemic dysregulation is not fully elucidated, there is compelling evidence implicating insulin resistance [42, 43]. Among the constellation of effects in an insulin resistant state is reduced suppression of lipolysis in adipose tissue, leading to a high free fatty acid influx and increased hepatic very low density lipoprotein (VLDL) secretion, thus causing hypertriglyceridemia and reduced plasma levels of high density lipoprotein (HDL) cholesterol [44].

## 5. Conclusion

In this population of people living with diabetes, high prevalence of metabolic syndrome exists. Gender vulnerability to metabolic syndrome and multiple cluster components are tilted towards the female subpopulation with diabetes.

## Figures and Tables

**Table 1:** Sociodemographic characteristics of the population under study stratified by gender.

Parameter	Total	Male	Female
Total respondents	162 (100)	61 (37.65)	101 (62.35)
Age	56.42 ± 10.64	55.93 ± 11.25	56.71 ± 10.29
Marital status			
Married	119 (73.46)	56 (91.80)	63 (62.38)
Single	43 (26.54)	5 (8.20)	38 (37.62)
Education background			
None	10 (6.17)	1 (1.64)	9 (8.91)
Basic	31 (19.14)	8 (13.11)	23 (22.77)
Secondary	69 (42.59)	21 (34.43)	48 (47.53)
Tertiary	52 (32.10)	31 (50.82)	21 (20.79)
Occupation			
None	49 (30.82)	16 (27.59)	33 (32.67)
Informal	69 (43.40)	20 (34.48)	49 (48.52)
Formal	41 (25.78)	22 (37.93)	19 (18.81)
Salt intake			
None	131 (82.39)	46 (79.31)	85 (84.16)
Moderate	25 (15.72)	9 (15.52)	16 (15.84)
High	3 (1.89)	3 (5.17)	0 (0.00)
Sugar intake			
None	127 (80.89)	42 (72.41)	85 (85.86)
Moderate	4 (2.55)	2 (3.45)	2 (2.02)
High	26 (16.56)	14 (24.14)	12 (12.12)
Fat intake			
None	137 (86.71)	52 (89.65)	85 (85.00)
Moderate	10 (6.33)	2 (3.45)	8 (8.00)
High	11 (6.96)	4 (6.90)	7 (7.00)

Data are presented as frequency with percentage in parenthesis or mean ± standard deviation.

**Table 2:** Anthropometric parameters of patients with type 2 diabetes seeking care at the Ho Municipal Hospital stratified by gender.

Parameter	Total	Male	Female	*p* value
Weight (Kg)	73.08 ± 14.26	71.59 ± 13.5	73.98 ± 14.69	0.3040
Height (m)	1.64 ± 0.08	1.69 ± 0.07	1.60 ± 0.08	<0.0001
BMI (Kg/m^2^)	27.46 ± 5.88	25.09 ± 4.55	28.89 ± 6.15	<0.0001
WC (cm)	93.52 ± 13.33	89.03 ± 10.01	96.24 ± 14.35	0.0014
HC (cm)	102.67 ± 15.48	98.79 ± 14.85	105.02 ± 15.45	0.0135
WHR	0.92 ± 0.08	0.91 ± 0.09	0.92 ± 0.07	0.5492
WHtR	0.57 ± 0.09	0.53 ± 0.06	0.60 ± 0.1	<0.0001
CI	1.29 ± 0.11	1.26 ± 0.09	1.30 ± 0.11	0.0140
AVI	18.00 ± 5.07	16.28 ± 3.51	19.04 ± 5.58	0.0017
BAI	31.39 ± 8.91	27.15 ± 7.69	33.94 ± 8.64	<0.0001
VAI	1.62 (0.30–7.89)	1.07 (0.30–6.01)	2.01 (0.42–7.89)	0.0231

Data are presented as mean ± standard deviation of the mean or median with minimum and maximum in parenthesis. BMI: body mass index, WC: waist circumference, HC: hip circumference, WHR: waist-to-hip ratio, WHtR: waist-to-height ratio, CI: Conicity Index, AVI: Abdominal Volume Index, BAI: Body Adiposity Index, and VAI: Visceral Adiposity Index.

**Table 3:** Haemodynamic and biochemical parameters of study population stratified by gender.

Parameter	Total	Male	Female	*p* value
SP	129.42 ± 20.07	129.72 ± 20.17	129.24 ± 20.11	0.8820
DP	79.15 ± 11.37	79.51 ± 11.46	78.93 ± 11.37	0.7550
FBG	7.35 ± 1.93	7.16 ± 2.12	7.47 ± 1.82	0.9870
Total cholesterol	4.90 ± 1.36	4.49 ± 1.17	5.15 ± 1.41	0.0030
Triglyceride	1.11 (0.38–5.30)	0.97 (0.38–4.46)	1.23 (0.42–5.30)	0.0110
HDL	1.25 ± 0.38	1.25 ± 0.44	1.25 ± 0.34	0.9740
LDL	3.02 ± 1.07	2.71 ± 0.99	3.21 ± 1.07	0.0030
VLDL	0.51 (0.17–2.03)	0.44 (0.17–2.03)	0.56 (0.19–1.66)	0.0120

Data is presented as mean ± standard deviation of the mean and median with minimum and maximum in parenthesis. SP: systolic blood pressure, DP: diastolic blood pressure, FBG: fasting blood glucose, HDL: high density lipoprotein, LDL: low density lipoprotein, and VLDL: very low density lipoprotein.

**Table 4:** Prevalence of metabolic syndrome and metabolic score among the population stratified by gender.

Parameters	Total	Male	Female	*p* value
	*N* = 162	*N* = 61	*N* = 101	
Prevalence of metabolic syndrome				
WHO	103 (63.58)	32 (52.46)	71 (70.30)	0.0285
NCEP-ATP III	71 (43.83)	26 (42.62)	45 (44.55)	0.8708
IDF	112 (69.14)	24 (39.34)	88 (87.13)	<0.0001
Prevalence of clustering of one or more components of metabolic syndrome				
WHO				
1	10 (6.17)	6 (9.84)	4 (3.96)	0.7306
2	49 (30.25)	23 (37.70)	26 (25.74)	
>2 without MetS	0 (0.00)	0 (0.00)	0 (0.00)	
NCEP-ATP III				
1	13 (8.02)	8 (13.12)	5 (4.95)	0.1208
2	78 (48.15)	27 (44.26)	51 (50.50)	
>2 without MetS	0 (0.00)	0 (0.00)	0 (0.00)	
IDF				
1	50 (30.86)	37 (60.66)	13 (12.87)	<0.0001
>2 without MetS	0 (0.00)	0 (0.00)	0 (0.00)	

Data is presented as frequency with corresponding percentage in parenthesis. WHO: World Health Organization, NCEP-ATP III: National Cholesterol Education Program, Adult Treatment Panel III, IDF: International Diabetes Federation, and MetS: metabolic syndrome. *p* is significant at 0.05.

**Table 5:** Prevalence of the various components of metabolic syndrome among the study population stratified by gender.

Parameter	Total	Male	Female	*p* value
NCEP-ATP III				
Abdominal obesity	78 (48.15)	5 (8.20)	73 (72.28)	<0.0001
Raised TG	27 (16.67)	4 (6.56)	23 (22.77)	0.0083
Reduced HDL	38 (23.46)	15 (24.59)	23 (22.77)	0.8491
High blood pressure	108 (66.67)	39 (63.93)	69 (68.32)	0.6079
WHO				
Central obesity	50 (30.86)	8 (13.11)	42 (41.58)	0.0001
Raised TG	27 (16.67)	4 (6.56)	23 (22.77)	0.0083
Reduced HDL	40 (24.69)	17 (27.87)	23 (22.77)	0.5731
High blood pressure	102 (62.96)	36 (59.02)	66 (65.35)	0.5021
IDF				
Abdominal obesity	112 (69.14)	24 (39.34)	88 (87.13)	<0.0001
Raised TG	27 (16.67)	4 (6.56)	23 (22.77)	0.0083
Reduced HDL	77 (47.53)	24 (39.34)	53 (52.48)	0.1437
High blood pressure	108 (66.67)	39 (63.93)	69 (68.32)	0.6079

Data is presented as frequency with corresponding percentage in parenthesis. TG: triglyceride, HDL: high density lipoprotein, WHO: World Health Organization, NCEP-ATP III: National Cholesterol Education Program, Adult Treatment Panel III, and IDF: International Diabetes Federation. *p* is significant at 0.05.
